# Neurological impairment and hyperglycemia following acute copper sulfate poisoning: a case report

**DOI:** 10.3389/fmed.2026.1758345

**Published:** 2026-03-10

**Authors:** Xiuqi Zhu, Huabin Wang, Bingqiang Su, Yelin Lou

**Affiliations:** 1 Department of Critical Care Medicine, Jinhua Hospital Affiliated to Zhejiang University School of Medicine, Jinhua, Zhejiang, China; 2 Department of Clinical Laboratory, Jinhua Hospital Affiliated to Zhejiang University School of Medicine, Jinhua, Zhejiang, China; 3 Department of Ultrasound Medicine, Jinhua Hospital Affiliated to Zhejiang University School of Medicine, Jinhua, Zhejiang, China

**Keywords:** convulsion, copper poisoning, hyperglycemia, neurological impairment, plasma exchange

## Abstract

**Rationale:**

Generally, clinical manifestations of copper sulfate intoxication include acute gastroenteritis, hemolytic anemia, and hepatorenal failure. Herein, we reported the case of a patient who developed neurological impairment and hyperglycemia following massive oral ingestion of copper sulfate. The article was to characterize these uncommon complications that extend beyond the typical toxicological spectrum, thereby enhancing the clinical understanding of copper sulfate toxicity.

**Patient concerns:**

A 14-year-old girl ingested approximately 50 grams of copper sulfate. Following initial resuscitation at a local hospital, she developed rapidly progressive tachypnea and hypoxemia. She was immediately transferred to Jinhua Central Hospital for further treatment.

**Diagnosis:**

The patient was diagnosed with acute severe copper sulfate intoxication, complicated by hemolytic anemia, hepatorenal failure, and two rare sequelae: neurological impairment and hyperglycemia.

**Interventions and outcomes:**

The patient received gastric lavage with 15,000 mL of isotonic solution at a local hospital. Due to the subsequent deterioration of respiratory function (tachypnea and hypoxemia), she was transferred to our hospital. Based on experience from previously reported successful cases of copper sulfate poisoning, we immediately implemented a comprehensive therapeutic regimen, including plasma exchange, copper chelation therapy (sodium dimercaptopropane sulfonate combined with calcium disodium edetate), and continuous renal replacement therapy. During hospitalization, the patient developed epileptic seizures, lower extremity neurological impairment, and persistent hyperglycemia. After symptomatic treatment with neurotrophic agents and insulin for glycemic control, clinical manifestations improved, with no recurrence of epilepsy. However, hyperglycemia and lower extremity neurological dysfunction showed only partial remission and were not fully resolved at discharge.

**Lessons:**

Acute massive copper sulfate poisoning may induce neurological impairment and hyperglycemia. Importantly, early monitoring and heightened vigilance for the delayed onset and protracted course of such complications are imperative.

## Introduction

Copper is an essential trace element in the human body, acting as a critical cofactor for numerous enzymes and contributing to multiple physiological processes. Copper sulfate has been historically used as an emetic, antifungal agent, and anthelmintic due to its unique pharmacological properties (1), but excessive oral ingestion can cause severe poisoning (2). Literature reports indicate that oral ingestion of 10 to 20 grams of copper sulfate may be fatal (1, 2).

Notably, its specific toxic effects on the nervous system. The early stage of copper sulfate poisoning is mostly characterized by non-specific gastrointestinal symptoms such as nausea, vomiting, and abdominal pain, and the condition can rapidly progress to life-threatening complications including gastrointestinal mucosal erosion, intravascular hemolysis, acute hepatorenal failure, and methemoglobinemia, with a high mortality rate (3). A survey of 123 cases of copper sulfate poisoning in Bangladesh showed that the main complications included acute liver injury (46 cases, 20 deaths), gastrointestinal bleeding (8 cases, 1 death), acute renal failure (5 cases, 3 deaths), acute intravascular hemolysis (5 cases, 2 deaths), and circulatory failure (3 cases, 2 deaths), with an additional 2 cases of unknown cause of death (4).

Nervous system and pancreatic endocrine function have rarely been thoroughly documented. This case report describes a successfully managed case of acute copper sulfate intoxication following oral ingestion of 50 grams of the substance. What distinguishes this case is the development of two rare complications not typically associated with this intoxication: neurological impairment and hyperglycemia. To the best of our knowledge, this is the first case report documenting this unique combination of complications, with the aim of enhancing the clinical understanding of the full spectrum of copper sulfate toxicity.

## Case Report

A 14-year-old female with a previous healthy condition, no history of diabetes or other special diseases, and no high-risk factors such as obesity or insulin resistance, ingested approximately 50 grams of copper sulfate and immediately developed severe burning pain in the oral cavity and stomach.

Physical examination findings on admission to our hospital: Body temperature 36.6 °C, heart rate 147 beats per minute, respiratory rate 37 breaths per minute, blood pressure 134/110 mmHg, and blood oxygen saturation 87% after oxygen inhalation via a mask at 15 L/min. The patient was drowsy, with a regular heart rhythm, and no abnormal findings were noted on bilateral lung auscultation. Due to the presence of severe metabolic acidosis complicated by respiratory acidosis, endotracheal intubation, mechanical ventilation support, and sodium bicarbonate administration were initiated to reverse the acidosis (see Table 1).

**Table 1:** Treatment schedule.

Time course	Clinical symptoms and signs	Key laboratory/imaging findings	Therapeutic measures
Immediately after poisoning	Oral ingestion of 50 g copper sulfate; salivation with corner of mouth erosion; urinary incontinence	/	Local hospital: gastric lavage with 15,000 mL of isotonic solution
3 h after poisoning	Respiratory distress; hypoxemia (SpO₂ decreased);elevated blood glucose	/	Transferred to our hospital; oxygen inhalation via 15 L/min mask
5 h after poisoning	Somnolence; metabolic acidosis complicated by respiratory acidosis; elevated blood glucose; unstable vital signs	Blood copper: 15,660 ng/mL; gastric lavage fluid copper: 11,4,830 ng/mL; blood gas analysis indicated mixed acid–base disorders; multiple organ dysfunction	Endotracheal intubation + mechanical ventilation; sodium bicarbonate correction; transferred to ICU; continuous renal replacement therapy (CRRT) + plasma exchange + copper chelation therapy; maintenance of electrolyte and blood glucose balance
22 h after poisoning	Increased muscle tone in left upper extremity and right lower extremity; limb edema	Lower extremity vascular ultrasound/CT ruled out DVT and fracture; rhabdomyolysis and compartment syndrome (CLS) confirmed	Mannitol dehydration + albumin supplementation; methylene blue to correct methemoglobinemia; continuation of chelation + CRRT regimen
7 days after poisoning	Persistently increased muscle tone, numbness, and tremor in right lower extremity; aggravated limited joint movement	MRI of right calf showed diffuse swelling of muscle groups/fascia; electroneuromyography revealed complete injury of the right common peroneal/tibial nerves distal to the popliteal fossa	Addition of furaltadone; combined mecobalamin + vitamins B1/B6 for neurotrophic therapy; continuation of basic treatment
16 days after poisoning	Limb convulsions; loss of consciousness; severe hyperglycemia	Random blood glucose > 34 mmol/L (maximum 40 mmol/L); normal head CT; EEG showed increased slow waves in the right background	Intensive insulin therapy for glycemic control; correction of electrolyte disturbances; continuation of neurotrophic therapy
Half a month after poisoning	Persistent hyperglycemia without remission	HbA1c increased to 7.7% (normal on admission); insulin 24.39 pmol/L; anti-GAD antibody 145.54 IU/mL	Adjustment of insulin dosage; sustained intensive glycemic control; evaluation of pancreatic function and monitoring of relevant indicators
At discharge	No recurrence of epilepsy; edema relieved; residual limb dysfunction	Renal function and blood glucose recovered; electroneuromyography indicated complete peripheral nerve injury	Transferred to rehabilitation hospital; oral neurotrophic drugs; insulin for sustained glycemic control; development of rehabilitation program
2 months after discharge (follow-up)	One episode of sudden epilepsy; persistent right lower extremity motor dysfunction	Blood glucose and renal function returned to normal; anti-GAD antibody decreased to 50.56 IU/mL; partial improvement of nerve injury	Levetiracetam for antiepileptic therapy; discontinuation of insulin; continuation of rehabilitation and neurotrophic therapy

The patient was transferred to the intensive care unit (ICU) at 5 h post-poisoning. Reports of heavy metal toxicants in the patient’s blood and gastric juice showed the following: blood copper level was as high as 15,660 ng/mL and blood zinc level was 1,550 ng/mL; gastric juice copper level was as high as 114,830 ng/mL and gastric juice zinc level was 1,195.6 ng/mL. Moreover, laboratory tests confirmed multisystem involvement, including hemolytic anemia, acute hepatic and renal impairment, coagulation dysfunction, methemoglobinemia, rhabdomyolysis, and gastrointestinal bleeding. Based on literature experience regarding successful management of copper sulfate poisoning, our hospital formulated and implemented a multidisciplinary comprehensive treatment regimen for the patient, including plasma exchange, copper chelation therapy (calcium disodium edetate and dimercaptopropanesulfonic acid sodium), methylene blue for correcting methemoglobinemia, and continuous renal replacement therapy (CRRT).

At 22 h post-poisoning, the patient developed increased muscle tone in the left upper limb and right lower limb. Lower extremity vascular ultrasound and computed tomography (CT) examinations ruled out deep vein thrombosis (DVT) and bone fractures. After multidisciplinary consultation, the condition was considered to be caused by rhabdomyolysis and capillary leak syndrome (CLS). Following mannitol administration for dehydration and albumin supplementation, limb edema was alleviated to some extent.

On the 7th day post-poisoning, the patient exhibited persistent increase in muscle tone of the right lower limb, aggravated limitation of active movement of the right ankle and toe joints, accompanied by numbness on the dorsum of the right foot, and tremors in the middle and ring fingers of the left hand. Magnetic resonance imaging (MRI) of the right lower leg revealed diffuse swelling of the leg muscle groups and fascia; neuroelectrophysiological examinations clearly indicated “complete injury of the right common peroneal nerve and tibial nerve at the level distal to the popliteal fossa”. A consultation with the Department of Neurology and Orthopedics suggested toxic nerve injury directly caused by copper sulfate poisoning. Therefore, on the basis of the original treatment, fursultiamine was added, and mecobalamin and vitamins B1/B6 were continued for neurotrophic therapy. After the aforementioned treatment, the patient’s edema was alleviated to some extent, while the movement limitation showed no improvement.

On the 16th day post-poisoning, the patient developed recurrent limb convulsions, manifested as increased muscle tone and loss of consciousness during seizures, with a duration ranging from tens of seconds to 3 min. A random blood glucose test showed a value >34 mmol/L. Cranial computed tomography (CT) revealed no structural abnormalities, while ambulatory electroencephalography (EEG) showed increased background slow activity (more prominent on the right side). A multidisciplinary consultation held that the convulsions were related to severe electrolyte disturbance and toxic encephalopathy. After active glycemic control and symptomatic treatment (without the use of antiepileptic drugs), the patient had no recurrent convulsions during hospitalization in our hospital.

**Figure 1:**
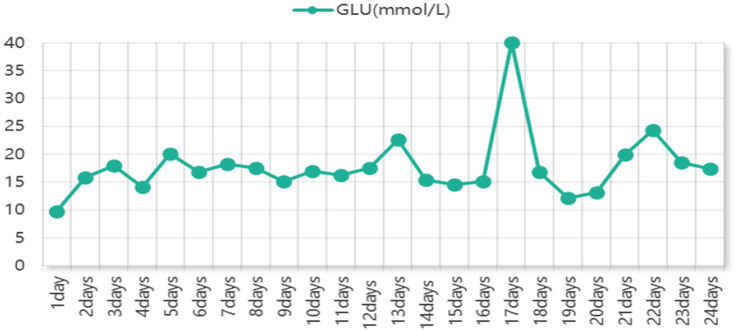
Blood glucose profile of the patient.

Throughout the hospitalization period, the patient consistently presented with significant hyperglycemia, with the highest level reaching 40 mmol/L (see Figure 1 for the blood glucose trend). Notably, the patient’s glycated hemoglobin (HbA1c) was within the normal range upon admission, but a re-examination half a month after poisoning showed an increase to 7.7%. Meanwhile, the insulin level was 24.39 pmol/L, the C-peptide level was 3.97 ng/mL, and the anti-glutamic acid decarboxylase antibody (anti-GAD antibody) was 145.54 IU/mL. A diagnosis of autoimmune diabetes secondary to immune dysfunction following poisoning was considered, and intensive insulin therapy was initiated accordingly.

**Figure 2:**
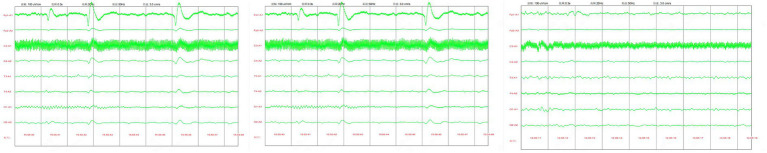
Ambulatory electroencephalogram (EEG).

Following aggressive treatment, the patient successfully survived the acute critical phase. However, renal function, blood glucose control, and right lower extremity nerve injury had not fully recovered at discharge, and the patient was transferred to another hospital for continued rehabilitation. At the 2-month follow-up after discharge, the patient experienced one episode of epilepsy. After treatment with levetiracetam 0.25 g orally twice daily, no further convulsions occurred, and no other neurological sequelae were observed. Blood glucose and renal function returned to normal, but motor dysfunction of the affected limb persisted; the patient is still undergoing rehabilitation therapy at present (see Figure 2).

## Discussion

As one of the essential trace elements in the human body, copper serves as a key cofactor for various enzymatic reactions. It is involved in core physiological processes such as oxygen transport, energy metabolism, cellular signal transduction, and bone marrow hematopoiesis, and also plays a crucial role in maintaining neuronal function and neurotransmitter synthesis in the central nervous system (5). Under physiological conditions, approximately 90% of copper binds to ceruloplasmin and is stored in hepatic lysosomes in a stable bound form (6). When copper ions exist in an unbound state, they generate harmful reactive oxygen species (ROS) (7).

After copper sulfate poisoning, excessive free copper accumulates rapidly in intracellular compartments. Thus, it directly induces hepatocyte necrosis and subsequent biliary obstruction, leading to liver injury (8). Within 12–24 h post-poisoning, copper ions bind to the sulfhydryl groups on the erythrocyte membrane, reducing the level of glutathione in erythrocytes and the activity of glucose-6-phosphate dehydrogenase (G6PD) (9). This impairs the stability of the erythrocyte membrane and triggers intravascular hemolysis. Meanwhile, as an oxidizing agent, copper oxidizes the divalent iron in oxyhemoglobin to trivalent iron, resulting in methemoglobinemia. This reduces the oxygen-carrying capacity of hemoglobin (10) and exacerbates tissue hypoxia. At 3–4 days post-poisoning, the patient develops renal impairment due to multiple factors: the direct toxic effect of copper sulfate on renal tubular epithelial cells, hypovolemia caused by toxic stress, and obstruction of renal tubules by hemoglobin released from hemolysis and myoglobin produced from rhabdomyolysis (10).

Excessive copper intake may cause irreversible cellular damage and neurotoxicity (11). It plays a crucial role in the occurrence and progression of neurodegenerative diseases (12) and cognitive impairment (13). In animal models of chronic copper exposure, spatial memory was impaired in mice, accompanied by the selective loss of presynaptic protein synaptophysin 1 and postsynaptic density (PSD)-93/95 in the hippocampus (14). On the other hand, copper significantly reduced the total number of cells and the length of nerve fibers in the cerebellar cortex and deep cerebellar nuclei in a dose-dependent manner (15). Meanwhile, copper accumulation was observed in the cortex, striatum, and cerebellum, leading to cognitive impairment and decreased motor coordination in mice (13, 16).

Copper-induced oxidative stress is the primary mechanism underlying copper neurotoxicity (17–19). Copper is a key component in critical processes of normal metabolism in brain cells and in the formation of neurotransmitters (20, 21). During acute poisoning, free copper crosses the blood–brain barrier and enters the central nervous system, leading to increased levels of non-ceruloplasmin-bound copper (free copper) in the plasma, cortex, and hippocampus (22). Through redox cycling, copper ions induce the production of reactive oxygen species (ROS), including superoxide anions, hydrogen peroxide, and hydroxyl radicals. Oxidative stress occurs when the level of ROS exceeds the intracellular antioxidant defense mechanisms (7). Free copper triggers the explosive accumulation of ROS and the activation of calpain, inducing oxidative stress in neurons. This damages presynaptic proteins (e.g., synaptophysin 1) and postsynaptic density proteins (e.g., PSD-93/95), resulting in impaired synaptic transmission (14) and subsequent neuronal damage. Meanwhile, high concentrations of Cu^2+^ can induce the activation of the classical caspase-dependent apoptotic pathway (studies have confirmed that classical caspase-dependent cell death is triggered in severely damaged neurons exposed to only twice the concentration of copper (7)). It accelerates neuronal necrosis by upregulating pro-apoptotic genes such as p53 and Bax, and downregulating anti-apoptotic genes such as Bcl-2 (1). In addition, copper induces transcriptional changes in genes involved in intracellular signal transduction and cell death (p53, c-fos, Bcl-2, and Bax) in a dose-dependent manner (1). These genes play important regulatory roles in mediating intracellular signal transduction pathways and initiating cell death programmes (7).

Copper is also involved in irreversible and non-specific binding to sulfhydryl groups (-SH) in various proteins. This non-oxygen-dependent oxidation leads to the inactivation and/or structural damage of important enzymes (including [specific enzymes, if applicable]), and also impairs neuronal function and survival (23, 24). In addition to the direct cytotoxicity of copper ions, they can further exacerbate neuronal damage by significantly increasing zinc ion concentrations (25, 26).

In this case, the patient presented with recurrent seizures after intoxication. Dynamic electroencephalography revealed increased slow-wave activity, indicating damage to the central nervous system. At present, seizures have been well controlled with pharmacotherapy. The patient also had concurrent peripheral nerve injury, with electroneuromyography indicating a complete injury. Combined with edema of the affected limbs, increased muscle tone, and magnetic resonance imaging findings, the peripheral nerve injury is considered secondary to local swelling and compression resulting from rhabdomyolysis.

Furthermore, although there are no reports of copper sulfate directly causing peripheral nerve injury, the neurotoxicity of copper in the central nervous system has been well documented. A direct toxic effect on peripheral nerves remains speculative and warrants further investigation. Importantly, causality cannot be inferred from a single case, and confounding factors such as rhabdomyolysis-related compression neuropathy should be considered.

Following poisoning, this patient experienced multiple epileptic seizures. Dynamic electroencephalography revealed increased slow-wave activity, indicating central nervous system impairment. At present, seizures have been well controlled with pharmacotherapy.

In addition, although there have been no previous reports of copper sulfate directly inducing peripheral nerve injury, the neurotoxicity of copper to the central nervous system has been well documented, and copper ions can promote cell survival (23, 24). Besides the direct cytotoxicity of copper ions, they can further exacerbate neuronal damage by significantly elevating zinc ion concentrations (25, 26). Therefore, a direct toxic effect on peripheral nerves cannot be excluded, and this issue warrants further investigation.

This patient had no prior history of diabetes mellitus, and glycated hemoglobin (HbA1c) was within the normal range on admission. Persistent hyperglycemia developed after poisoning, with blood glucose repeatedly exceeding 34 mmol/L (changes in blood glucose after admission are shown in Figure 1). The underlying mechanisms may include two aspects:

First, the stress response induced by poisoning leads to elevated blood glucose (27). Poisoning-related stress activates the sympathetic-adrenal axis, resulting in increased secretion of hyperglycemic hormones such as glucagon and cortisol. Second, copper ions cause pancreatic injury. On the one hand, copper ions can induce autophagy of pancreatic *β*-cells via the adenosine 5′-monophosphate-activated protein kinase/mammalian target of rapamycin (AMPK/mTOR) signaling pathway, disrupt metabolic pathways, and impair glucose metabolism (28). On the other hand, copper ions promote the formation of high-molecular-weight covalent aggregates of glutamic acid decarboxylase (GAD) in pancreatic *β*-cells, impairing insulin synthesis and secretion (29), which is consistent with the clinical finding of elevated anti-GAD antibodies in this patient.

A study by Trigwell et al. (29) confirmed that in islet cells surviving treatment with copper sulfate and hydrogen peroxide, copper ions can continuously generate reactive oxygen species (ROS) through the Fenton reaction. The generated ROS specifically act on glutamic acid decarboxylase, leading to the formation of high-molecular-weight covalently linked aggregates. The patient received continuous copper chelation therapy during hospitalization. Follow-up revealed that after transfer for rehabilitation and discontinuation of the chelating agent, blood glucose returned to normal and insulin was discontinued. Repeated measurement showed that anti-glutamic acid decarboxylase antibody decreased to 50.56 IU/mL.

The above clinical outcome may be associated with the discontinuation of copper chelation therapy. Mechanistically, free metals can catalyze the generation of reactive oxygen species. However, existing studies (30) have shown that the use of exogenous metal chelators may instead enhance the production of reactive oxygen species, thereby exacerbating pancreatic *β*-cell injury. This mechanism is consistent with the clinical observation of improved blood glucose after chelator withdrawal. Nevertheless, the dose–response relationship of this effect in clinical practice has not been clearly established.

To the best of our knowledge, this is the first reported case of copper sulfate poisoning complicated with neurological impairment and hyperglycemia. It reminded us that in the clinical management of such patients, we need to be vigilant about pancreatic and neurological damage after poisoning, conduct relevant examinations as early as possible, detect these rare complications in a timely manner, and improve the patient’s prognosis.

## Data Availability

The original contributions presented in the study are included in the article/supplementary material, further inquiries can be directed to the corresponding author.
